# Effect of Centhaquine on the Coagulation Cascade in Normal State and Uncontrolled Hemorrhage: A Multiphase Study Combining Ex Vivo and In Vivo Experiments in Different Species

**DOI:** 10.3390/ijms25063494

**Published:** 2024-03-20

**Authors:** Athanasios Chalkias, Gwendolyn Pais, Anil Gulati

**Affiliations:** 1 Institute for Translational Medicine and Therapeutics, University of Pennsylvania Perelman School of Medicine, Philadelphia, PA 19104-5158, USA; 2 Outcomes Research Consortium, Cleveland, OH 44195, USA; 3 Chicago College of Pharmacy, Midwestern University, Downers Grove, IL 60515, USAanil.gulati@pharmazz.com (A.G.); 4 Pharmazz Inc., Research and Development, Willowbrook, IL 60527, USA; 5 Department of Bioengineering, The University of Illinois at Chicago, Chicago, IL 60607, USA

**Keywords:** coagulation, shock, centhaquine, coagulopathy, critical care medicine, anesthesiology, resuscitation, vasopressor, translational research

## Abstract

Centhaquine is a novel vasopressor acting on α2A- and α2B-adrenoreceptors, increasing venous return and improving tissue perfusion. We investigated the effects of centhaquine on blood coagulation in normal state and uncontrolled hemorrhage using ex vivo and in vivo experiments in different species. Thromboelastography (TEG) parameters included clotting time (R), clot kinetics [K and angle (α)], clot strength (MA), and percent lysis 30 min post-MA (LY30). In normal rat blood, centhaquine did not alter R, K, α, MA, or LY30 values of the normal vehicle group or the antithrombotic effects of aspirin and heparin. Subsequently, New Zealand white rabbits with uncontrolled hemorrhage were assigned to three resuscitation groups: Sal-MAP 45 group (normal saline to maintain a mean arterial pressure, MAP, of 45 mmHg), Centh-MAP 45 group (0.05 mg kg^−1^ centhaquine plus normal saline to maintain a MAP of 45 mmHg), and Sal-MAP 60 group (normal saline to maintain a MAP of 60 mmHg). The Sal-MAP 45 group was characterized by no change in R, reduced K and MA, and increased α. In the Centh-MAP 45 group, TEG showed no change in R, K, and α compared to saline; however, MA increased significantly (*p* = 0.018). In the Sal-MAP 60 group, TEG showed no change in R, an increase in α (*p* < 0.001), a decrease in K (*p* < 0.01), and a decrease in MA (*p* = 0.029) compared to the Centh-MAP 45 group. In conclusion, centhaquine does not impair coagulation and facilitates hemostatic resuscitation.

## 1. Introduction

Patients with severe hemorrhage may experience a significant depletion of intravascular volume. This is initially compensated by various regulatory mechanisms including activation of the sympathetic nervous system and immediate release of catecholamines, the concentration of which may increase up to 40-fold [1,2]. In later stages of hemorrhagic shock, loss of compensatory mechanisms to maintain arterial blood pressure and cardiac output is usually due to decreased cardiac function and failure to maintain sympathetically induced vasoconstriction [3].

A key factor in the pathophysiology of hemorrhagic shock is the development of trauma-induced coagulopathy (TIC) [4]. Several mechanisms have been proposed to explain the development of these abnormal coagulation processes, but the heterogeneity in injuries and patient profiles makes it difficult to define a dominant mechanism. In general, early and late TIC results from the collective insults of tissue injury, shock, and traumatic brain injury, as well as individual responses to these insults [5]. Trauma-induced coagulopathy can be characterized by hypo- or hypercoagulability. The former may occur due to hyperfibrinolysis, platelet dysfunction, depletion of fibrinogen, and/or decreased thrombin generation. The latter is characterized by increased thrombin generation, hyperfibrinogenemia, platelet activation, and/or fibrinolysis shutdown. Of note, the mechanisms underlying the various phenotypes (bleeding, thrombotic, or mixed) can occur at different times after injury; therefore, many TIC phenotypes change over time [4,5,6].

Although the role of exogenous catecholamines in hemorrhagic shock is controversial, norepinephrine, the currently recommended first-choice vasopressor, is usually started when blood pressure cannot be maintained despite initial fluid expansion [2,7]. Nevertheless, in patients with severe hypovolemia, the blood vessels are already maximally constricted and exogenous catecholamines will probably aggravate tissue hypoperfusion [8,9]. In addition, catecholamines can have numerous other biological effects as a result of the ubiquitous presence of their receptors, including disturbances in energy metabolism, mitochondrial function, immune response, and organ system homeostasis [10]. Moreover, human data strongly indicate that catecholamines affect hemostasis and the coagulation–fibrinolysis balance via combined α2- and β2-adrenoreceptor stimulation [10,11]. Actually, the fact that sympathetic nervous system overactivity and elevated catecholamine levels hasten blood clotting in animals and humans has been known for more than a century [11,12,13]. However, whether alternative compounds are superior to catecholamines in improving tissue perfusion without affecting coagulation remains unknown.

Centhaquine (2-[2-(4-(3-methyphenyl)-1-piperazinyl)]ethyl-quinoline) citrate is a novel vasopressor acting on α2B-adrenoreceptors which are abundantly present in veins while scarcely present in arteries, contributing most to venoconstriction [14]. It also activates central α2A-adrenoreceptors, reducing systemic vascular resistance and afterload by attenuating sympathetic outflow. Therefore, centhaquine increases venous return and cardiac output while facilitating tissue perfusion. This is confirmed by several experimental studies and clinical trials, which showed the superior effectiveness of centhaquine compared to commonly used resuscitative agents in reducing mortality following hemorrhagic shock [14,15,16,17]. Of note, centhaquine is the only late-stage clinical developmental drug that has demonstrated an 8.8% absolute reduction in mortality after hypovolemic shock.

Two multicenter, randomized controlled trials investigating the safety and efficacy of centhaquine in patients with hypovolemic shock showed that hematological and conventional coagulation parameters (platelet count, prothrombin time, fibrinogen value, and international normalized ratio) did not differ significantly between the centhaquine and control groups [16,17]. However, it should be considered that conventional measures of coagulation may represent distinct mechanisms independent of biochemical clot strength [18] and also overestimate TIC in stable trauma patients [19,20]. On the other hand, thromboelastography (TEG) can account for the balance of coagulation—taking into account both clotting factors and endogenous anticoagulant proteins—and provide insights into clot formation, clot stability through platelet–fibrin interactions, and clot retraction and/or lysis [21]. As correlations between TEG parameters and standard laboratory results may not be perfect [22,23], especially in critically ill patients, an effect of centhaquine on blood coagulation remains possible.

The aforementioned clinical observations motivated us to follow a bedside-to-bench approach and further investigate the effects of centhaquine on the coagulation cascade in the laboratory. In the present study, we evaluated the potential effects of centhaquine on blood coagulation in normal state and severe hemorrhage using ex vivo and in vivo experiments in different species.

## 2. Results

### 2.1. Effect of Centhaquine on Coagulation by Ex Vivo Thromboelastography (Rat Model)

Thromboelastography parameters of the normal vehicle-treated group were found to be as follows: R = 2.9 ± 0.2 min, K = 0.96 ± 0.0 min, α = 76.0 ± 0.7°, MA = 71.2 ± 0.7 mm, and LY30 = 0.14 ± 0.0%. The values did not differ when water:PEG 400 4:1 *v/v* was used as a vehicle: R = 2.9 ± 0.2 min, K = 0.9 ± 0.1 min, α = 77.9 ± 1.3°, MA = 74.6 ± 1.8 mm, and LY30 = 0.3 ± 0.1%. Centhaquine did not alter the R, K, α, MA, or LY30 values of vehicle-treated groups.

**Figure 1:**
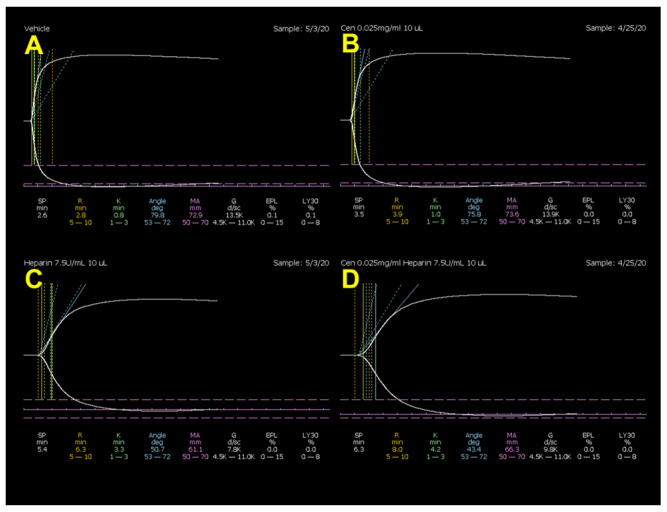
Representative coagulation tracings from the thromboelastograph in normal rats from (**A**) vehicle-, (**B**) centhaquine-, (**C**) heparin-, and (**D**) centhaquine + heparin-treated groups.

**Figure 2:**
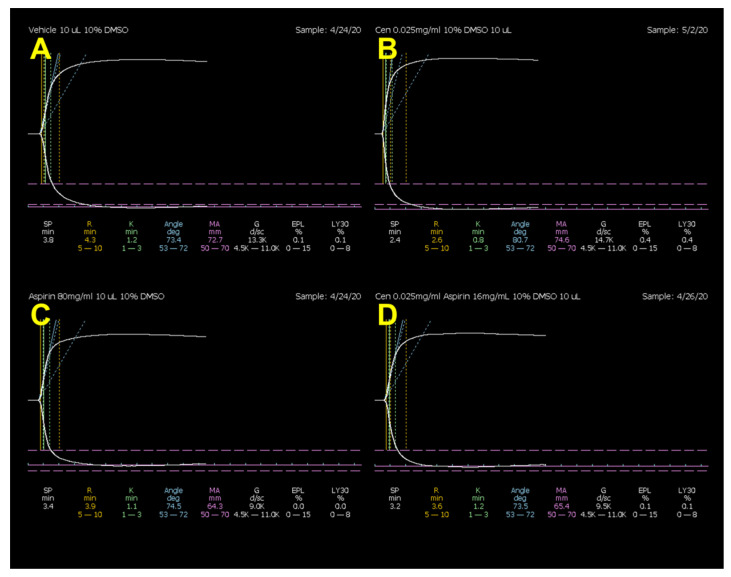
Representative coagulation tracings from the thromboelastograph in normal rats from (**A**) 0.3% DMSO-, (**B**) centhaquine-, (**C**) aspirin-, and (**D**) centhaquine + aspirin-treated groups.

Heparin did not produce any change in LY30 values, but significantly increased R by 56% and K by 64% and decreased α by 32% and MA by 11%. Centhaquine did not affect the heparin-induced increase in R and K or the decrease in α and MA (Figure 1 and Table 1). Aspirin did not produce any change in R, K, α, or LY30 values, but produced a 16% decrease in MA. Centhaquine did not produce any change in aspirin-induced decrease in MA (Figure 2 and Table 2). DMSO (0.3%) did not produce any effect on TEG parameters (Table S1).

### 2.2. Development of a Translational Rabbit Model of Uncontrolled Hemorrhagic Shock

Heart rate decreased significantly with hemorrhage in all groups (*p* < 0.001). Subsequently, it increased with resuscitation at 60 min and 120 min in Group 2 and Group 3. However, heart rate in Group 3 at 60 min and 120 min was slightly lower compared to saline (Figure S1). Mean arterial pressure decreased significantly with hemorrhage in all groups (*p* < 0.001, except group 3 where *p* = 0.003) and increased significantly with resuscitation at 60 min and 120 min (Figure S2, Table S2).

Blood loss was not significantly different among groups (Group 1: 44.4 ± 6.6 mL; Group 2: 42.5 ± 2.5 mL; Group 3: 36.4 ± 2.5 mL; *p* = 0.203; Figure S3), while, across all groups, lactate levels increased significantly with hemorrhage (*p* < 0.001). As expected, resuscitation significantly decreased lactate at 60 min and 120 min (Figure S4, Table S3; *p* < 0.001). Animals in the centhaquine resuscitation group (Group 3) required significantly less infusion volume compared to normal saline animals (Group 2) (133.9 ± 10.8 mL vs. 195.1 ± 10.7 mL, *p* < 0.001) to maintain MAP at 45 mmHg for 60 min (Figure S5).

Fluctuations of body temperature were similar in saline and centhaquine treated groups. Pre-experimental body weight was nearly identical across groups (Group 1: 2.7 ± 0.08 kg; Group 2: 2.7 ± 0.05 kg; Group 3: 2.8 ± 0.06 kg). The wet-to-dry ratio in Group 2 (5.45 ± 0.15) and Group 3 (5.56 ± 0.16) was similar; however, both were significantly greater (*p* = 0.0028) than the wet-to-dry ratio in Group 1 (4.50 ± 0.08) (Figure S6).

The overall mortality of animals subjected to hemorrhagic shock was 16% (five of 30 animals); all five deaths occurred in Group 1.

### 2.3. Effect of Hypotensive Resuscitation with Centhaquine Versus a Higher Blood Pressure Target on Blood Coagulation

**Figure 3:**
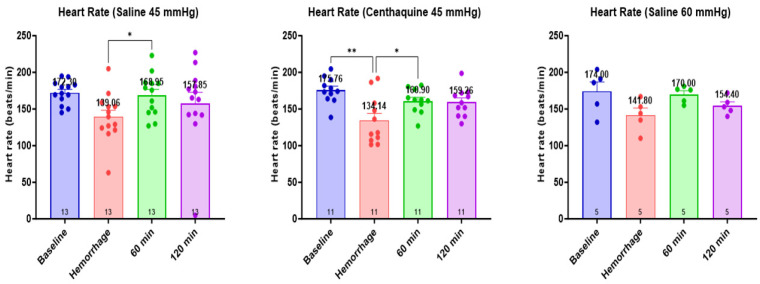
Fluctuation of heart rate during the experiment. * 0.01 ≤ *p* < 0.05; ** 0.001 ≤ *p* < 0.01.

**Figure 4:**
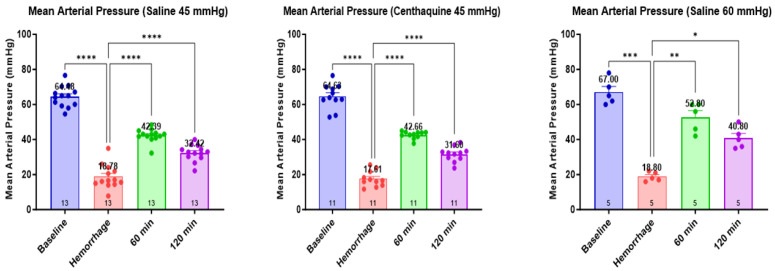
Fluctuation of mean arterial pressure during the experiment. * 0.01 ≤ *p* < 0.05; ** 0.001 ≤ *p* < 0.01; *** 0.0001 ≤ *p* < 0.001; **** *p* < 0.0001.

The heart rate was significantly lower at the end of the hemorrhage in all groups (*p* < 0.01). Subsequently, it increased with resuscitation at 60 min and 120 min in the Sal-MAP 45 group and the Centh-MAP 45 group (*p* < 0.01 for both) (Figure 3). Mean arterial pressure decreased significantly with hemorrhage in all groups (*p* < 0.0001, except Sal-MAP 60 group where *p* < 0.001) and increased significantly with resuscitation at 60 min and 120 min (Sal-MAP 45 group and Centh-MAP 45 group: *p* < 0.0001 for both time points; Sal-MAP 60 group: *p* < 0.01 at 60 min and 120 min) (Figure 4).

**Figure 5:**
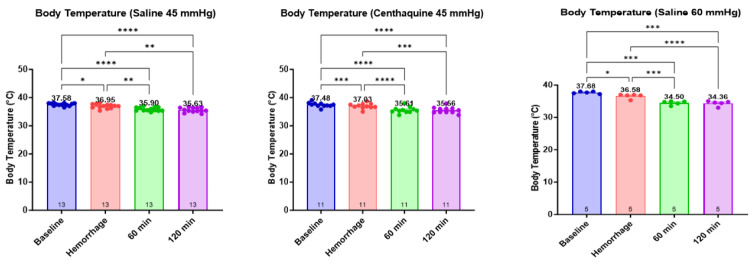
Change in body temperature during the experiment. * 0.01 ≤ *p* < 0.05; ** 0.001 ≤ *p* < 0.01; *** 0.0001 ≤ *p* < 0.001; **** *p* < 0.0001.

Significant differences were observed in body temperature between the three groups during hemorrhage (*p* < 0.001) and resuscitation at 60 min (*p* < 0.001) and 120 min (*p* < 0.001) (Figure 5). Lactate increased significantly during hemorrhage in all groups (Sal-MAP 45 group: *p* = 0.013; Centh-MAP 45 group and Sal-MAP 60 group: *p* < 0.001). Subsequently, it decreased with resuscitation at 60 min and 120 min in the Centh-MAP 45 group (*p* < 0.01 for both time points) and the Sal-MAP 60 group (*p* < 0.001 for both time points).

**Figure 6:**
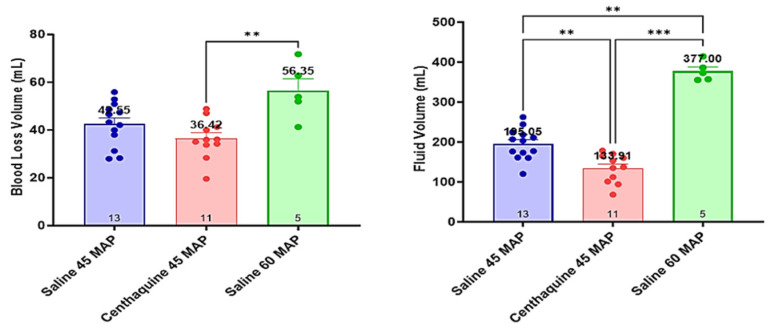
Differences in blood loss and fluid volume among groups. ** 0.001 ≤ *p* < 0.01; *** 0.0001 ≤ *p* < 0.001.

Blood loss was similar in the Sal-MAP 45 group and the Centh-MAP 45 group and was higher in Sal-MAP 60 group (*p* < 0.01). The Sal-MAP 45 group required a greater volume of saline to maintain MAP at 45 mmHg compared to the Centh-MAP 45 group (195 ± 9 mL vs. 134 ± 11 mL, *p* = 0.001). The amount of saline required to maintain the target MAP in the Sal-MAP 60 group was markedly greater compared to that required for the Sal-MAP 45 group (195 ± 9 mL vs. 377 ± 11 mL, *p* < 0.001) (Figure 6).

**Figure 7:**
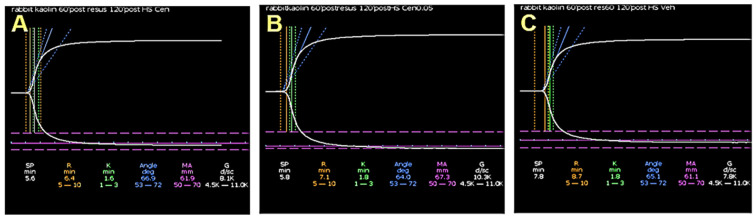
Representative coagulation tracings from the thromboelastograph in hemorrhaged rabbits from the Sal-MAP 45 group (**A**), Centh-MAP 45 group (**B**), and Sal-MAP 60 group (**C**) at 120 min showing increased clot strength with centhaquine resuscitation.

**Figure 8:**
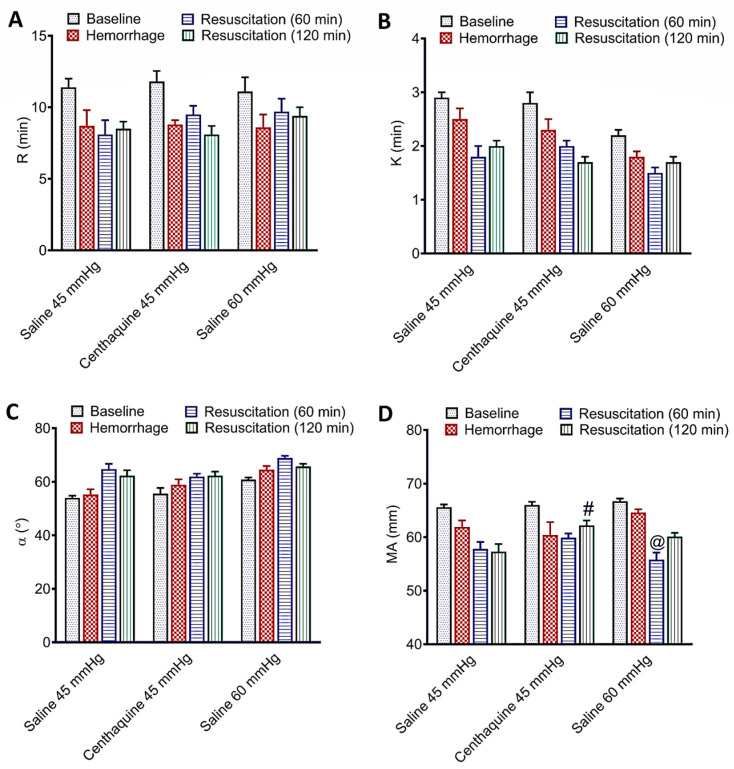
Effect of saline or centhaquine resuscitation on TEG parameters R (**A**), K (**B**), α angle (**C**), and MA (**D**) in hemorrhaged rabbits. Values are expressed as mean ± SEM. ^#^ *p* = 0.018 compared to saline (45 mmHg) resuscitation (Sal-MAP 45 group); ^@^ *p* = 0.029 compared to centhaquine resuscitation (Centh-MAP 45 group).

Baseline R, Κ, α, and MA were 11.4 ± 0.6 min, 2.9 ± 0.1 min, 54.0 ± 0.8°, and 65.6 ± 0.5 mm, respectively. Hemorrhagic shock produced a decrease in R, K, and MA, and an increase in α, but TEG parameters did not differ significantly among groups at the end of the hemorrhage. The Sal-MAP 45 group was characterized by no change in R, a decrease in K and MA, and an increase in α. In the Centh-MAP 45 group, TEG showed no change in R, K, and α compared to saline; however, MA increased significantly (*p* = 0.018). In the Sal-MAP 60 group, TEG showed no change in R, an increase in α (*p* < 0.001), a decrease in K (*p* < 0.01), and a decrease in MA (*p* = 0.029) compared to centhaquine resuscitation (Figure 7 and Figure 8).

## 3. Discussion

Our ex vivo experiments revealed that centhaquine did not alter the TEG parameters of the normal vehicle group or the antithrombotic effects of aspirin and heparin on blood collected from healthy rats. In the rabbit model of hemorrhagic shock, centhaquine resuscitation to MAP 45 mmHg (Centh-MAP 45 group) did not change the duration required for blood to begin clotting (R), the duration needed for the clot to reach a specific level of firmness (K), and the rapidity of fibrin build-up and cross-linking (α) compared to saline. However, it significantly increased the ultimate strength and firmness of the clot (MA). Additional saline to maintain MAP 60 mmHg (Sal-MAP 60 group) significantly increased the rapidity of fibrin build-up and cross-linking (α), decreased the duration needed for the clot to reach a specific level of firmness (K), and decreased the strength and firmness of the clot (MA) compared to centhaquine. These findings indicate that centhaquine does not affect blood coagulation under normal conditions (steady state), while in severe hemorrhage it does not adversely affect coagulation and increases clot strength compared to saline.

Uncontrolled post-traumatic bleeding has been reported to cause 25% of all injury-related deaths and 40–80% of potentially preventable early traumatic deaths [24]. The fact that several mechanisms are implicated in the development of abnormal coagulation processes, often not recognized during trauma resuscitation, contributes particularly to the increased mortality. Of note, TIC may be present even before the onset of resuscitation and correlates with the severity of trauma. This must be taken into account during clinical practice, especially when managing fragile individuals or those with comorbidities. Also, an increasing number of patients are treated with oral antiplatelet agents, anticoagulants, or combined therapy [25,26]; in these individuals, residual antithrombotic effects may contribute to excessive bleeding.

The clinical efficacy of centhaquine as a resuscitation agent has been demonstrated in two randomized trials in patients with hypovolemic shock [16,17]. Of note, in these studies, conventional hematological and coagulation parameters did not differ significantly between the centhaquine and control groups [16,17]. Although these results were encouraging, centhaquine effects on blood coagulation have continued to remain elusive until now. Our translational experiments in two different species show that centhaquine does not affect coagulation in normal and bleeding conditions. Instead, it increases clot strength and reduces blood loss and fluid requirements during resuscitation of hemorrhagic shock.

It remains unknown whether centhaquine effects on blood coagulation are due to its histochemical form per se, its hemodynamic effects, or both. However, our findings are very important, as early TIC (less than 6 h) usually occurs due to inadequate hemostatic control and thrombus formation in massively hemorrhaging patients [24]. A major contribution to these phenomena is attributed to the excessive administration of crystalloids, which increases the risk of hemodilution and other complications including acute lung injury/acute respiratory distress syndrome, hypothermia, infections, intra-abdominal hypertension/abdominal compartment syndrome, multiple organ failure, and death [4]. Therefore, the use of centhaquine may facilitate the implementation of the latest Advanced Trauma Life Support guidelines, which include less stringent suggestions for crystalloid administration [27,28], enhance clot stabilization, one of the first treatment goals once the TIC is identified [29], decrease the incidence of complications, and improve outcomes [16,17]. To the best of our knowledge, centhaquine is the first vasopressor agent that does not impair coagulation and may facilitate hemostatic resuscitation.

In many institutions, during resuscitation of hypovolemic shock, exogenous catecholamines are usually started when blood pressure cannot be maintained despite initial fluid expansion or when blood/blood products are unavailable [2,7]. However, administration of vasopressors in severe hypovolemia is usually associated with the worsening of tissue perfusion and bleeding attributed to marked arterial vasoconstriction and disruption of unstable clots by higher pressures, respectively. Importantly, exogenous catecholamines may also impair hemostasis and the coagulation–fibrinolysis balance through vascular α and β endothelial adrenoreceptor stimulation [10,11].

The most studied catecholamine to date is epinephrine. A dose-dependent stimulation of factor VIII clotting activity, von Willebrand factor antigen, and tissue-type plasminogen activator has been observed within 15–40 min of the start of its infusion [11,30,31,32]. In addition, epinephrine may induce functionally active factor VIII from the spleen, as well as short-term recruitment and activation of platelets [33,34]. Of note is that epinephrine-induced rise in platelet count is not significantly influenced by alprenolol, a non-selective beta blocker as well as a 5-HT1A and 5-HT1B receptor antagonist [35].

Norepinephrine, the currently recommended first-choice vasopressor in hypotensive trauma patients, predominantly enhances α-adrenoceptor stimulation and has a mild effect on β1-adrenoceptors [36]. The β2-adrenergic effects of norepinephrine are less defined and may occur with high concentrations of this drug. In healthy individuals, elevation of circulating norepinephrine caused concentration-dependent platelet activation in vivo and, perhaps more interestingly, aspirin pretreatment only partly attenuated this effect [37]. In single-blind placebo-controlled trials, a 15-min norepinephrine infusion induced significantly greater increases over time in plasma levels of FVIII:C, fibrinogen, and D-dimer compared to the placebo (saline infusion), overall suggesting that norepinephrine induces a prothrombotic state [38]. Also, phentolamine attenuated the norepinephrine-induced increase in FVIII:C and D-dimer (but not fibrinogen) to a level that was not significantly different from the placebo condition, suggesting that α-adrenergic mechanisms may partly underlie norepinephrine effects on blood coagulation [38].

The aforementioned evidence suggests that catecholamines may affect coagulation through pathways other than those involving adrenergic receptors [38,39,40], possibly because of their histochemical form. Considering their frequency of use in daily clinical practice, it is almost certain that they negatively affect—at least in part—patient management and hemostatic resuscitation. Also, the prothrombotic effects mediated by catecholamines may enhance the change in a patient’s coagulation status from hypocoagulable to hypercoagulable [41]. In contrast, the present study shows that centhaquine does not impair coagulation in normal and bleeding conditions and may increase clot strength in hemorrhagic shock. As the coagulation profile is similar among mice, rabbits, and humans, our findings are clinically relevant and may improve survival in patients with hemorrhagic shock [42,43].

Based on the mechanism of action of centhaquine, another possible explanation for its favorable effects may be its shear-protective action [41]. Of note, catecholamines affect the entire vasculature and may cause abnormal arterial shear-induced responses in trauma patients. For example, catecholamine-induced shear stress may promote platelet–platelet and platelet–vessel wall interaction via several mechanisms, including combined α2- and β2-adrenoreceptor stimulation [44,45,46]. Considering that venous endothelial cells are less thrombogenic than arterial endothelial cells [47], centhaquine, as a pure venoconstrictor (α2B-adrenoreceptors), may maintain a steady flow and no or minimal pulsatility in veins [48,49], thus exploiting the beneficial anti-thrombogenic profile of venous endothelial cells [47] without directly affecting the arterial side of the circulation [41]. Moreover, its action on central α2A-adrenoreceptors facilitates arteriolar flow and tissue perfusion, which enhances organ protection during severe hemorrhage [50,51]. Further research is necessary to fully clarify the effects of centhaquine at the molecular and cellular level and, ultimately, its integrative mechanism of action.

We acknowledge a few limitations. This study was performed on apparently healthy animals without any known previous comorbidity. Also, our rabbit model did not take into account the associated trauma injuries or the possible effects of anesthesia. However, it simulates a clinically relevant scenario of uncontrolled hemorrhagic shock. Another limitation is that we included only male rabbits. However, distinctly different fibrinolytic system responses are observed among individual rabbits [51]. In addition, several similarities have been observed between “rat and human” and “rabbit and human” coagulation processes [43,52,53,54,55,56], strengthening the translational impact and clinical relevance of our findings. We also recognize that TEG is not a perfect assay and may lead to overtransfusion of blood products even when no longer indicated, increasing the risk of adverse events [57]. However, TEG-guided management may improve survival and is recommended for use in clinical practice [6]. Considering that centhaquine enhances thrombus formation and restoration of hemostatic balance, the combined use of centhaquine and TEG in clinical practice may lead to fewer transfusions in the early phase of resuscitation.

## 4. Materials and Methods

### 4.1. The Rationale for Choosing Translational Models and Ethics Approval

The present study used ex vivo and in vivo experiments to enhance the translational impact and clinical relevance of the research. We used mice, which are widely used to study hemostasis and blood coagulation disorders because their hemostatic system is similar to that of humans [43], and rabbits, which are larger animals and exhibit a coagulation profile similar to that of humans [42].

Animal care and use for experimental procedures were approved by the institutional animal care and use committee. The study was conducted according to the Guide for the Care and Use of Laboratory Animals and the principles of the 3Rs, which stands for Replacement, Reduction, and Refinement and represents a responsible approach to conducting more humane animal research (https://www.nc3rs.org.uk/the-3rs, accessed on 10 December 2023). This manuscript adheres to the applicable ARRIVE 2.0 guidelines [58].

### 4.2. Effects of Centhaquine on Thromboelastometry Analysis of Rat Whole Blood

An initial study was conducted to investigate the effect of centhaquine on the coagulation cascade and the antithrombotic effects of heparin and aspirin using TEG in whole blood collected from rats under normal (steady state) condition. Aspirin and heparin were used to further increase the translational impact and clinical relevance of our findings, considering their frequency of use and patient heterogeneity in real-world practice, as well as their effects on patient physiology.

#### 4.2.1. Animals

Male Sprague–Dawley rats weighing 275 g to 325 g (Harlan, Indianapolis, IN, USA) were used. Animals were allowed to acclimate for at least four days before being handled in a room with controlled temperature (23 ± 1 °C), humidity (50 ± 10%), and light (6:00 A.M. to 6:00 P.M.). Food and water were made available continuously.

The animals were anesthetized with urethane (Acros Organics, New Jersey, NJ, USA) in a 1.5 g Kg^−1^ body weight dose via intraperitoneal injection. Urethane was selected because it produces long-lasting (8–10 h) anesthesia with minimal cardiovascular and respiratory system depression. It also produces a level of surgical anesthesia characterized by the preservation of cardiovascular reflexes [59]. Anesthetized rats were immobilized on a surgical board equipped with a controlled heating pad, and the right common carotid artery was exposed through a midline incision for whole blood collection.

#### 4.2.2. Drugs and Experimental Protocol

In order to determine the effect of centhaquine on coagulation, we compared the effect of centhaquine with vehicle (normal saline) on TEG. In addition, we assessed the effect of centhaquine on heparin-induced inhibition of coagulation using a heparin plus vehicle and a centhaquine plus heparin group. Furthermore, we investigated the effect of centhaquine on the antithrombotic effect of aspirin using an aspirin plus vehicle and a centhaquine plus aspirin group. Since aspirin is dissolved in 0.3% DMSO, we also determined its effect on TEG.

Centhaquine citrate dihydrate (Pharmazz India Pvt. Ltd., Greater Noida, India) was dissolved in 0.9% normal saline solution or water–polyethylene glycol 400 (Carbowax^®^, Fisher Scientific, Fair Lawn, NJ, USA) (4:1, *v*/*v*) and diluted to obtain a final concentration of 0.00027 mg centhaquine per 360 μL of citrated whole blood. The concentration of centhaquine was selected based on the recommended dose in hemorrhagic shock (0.05 mg kg^−1^) [60].

Heparin sodium injection USP (APP Pharmaceuticals, LLC, Schaumburg, IL, USA) was mixed with 0.9% normal saline solution to obtain a concentration of 0.075 Units per 360 μL citrated whole blood. Aspirin USP (PCCA, Houston, TX, USA) was dissolved in water:polyethylene glycol 400 (4:1, *v*/*v*) [61] through warming in a water bath to obtain a concentration of 0.362 mg per 360 μL of citrated whole blood. The concentrations of heparin sodium and aspirin were selected based on preliminary studies. The human dose was obtained from published literature and converted to the rat dose using the formula animal dose in mg kg^−1^ = human equivalent dose in mg kg^−1^ × 6.2 [62]. Using 20 mL as the blood volume of a 300 g rat as standard [63], the drug concentrations were calculated for the 360 μL capacity of the TEG cup.

#### 4.2.3. Blood Collection

Arterial blood was obtained by cannulation of the carotid artery in rats. The first five drops of blood were discarded to drain off the sodium citrate solution from the tubing and to avoid excess tissue factor. Blood was then collected in a syringe in one-tenth volume of 3.2% (0.105 M) sodium citrate (pH 7.4) and transferred to a polyethylene tube. The tube was capped and gently inverted three times to mix the blood with the citrate.

#### 4.2.4. Thromboelastography

TEG^®^ 5000 Hemostasis Analyzer with software version 4.2 (Haemonetics Corporation, Niles, IL, USA) was used for coagulation analyses. Official representatives of the manufacturer performed calibrations. Quality controls were performed once before the set of experiments using biological QC level I & II Kits (Haemonetics Corporation).

The cup, pin, and reagents were prepared, and the channel was programmed for citrated kaolin blood. The kaolin reagent was used to reduce variabilities and running time of a whole blood TEG sample. The kaolin reagent (Haemonetics Corporation) was warmed to room temperature and tapped to collect the liquid to the bottom of the vial. An excess of calcium was added to reverse the effects of citrate and enable the blood to clot again. Twenty microliters of 0.2 M calcium chloride (Haemonetics Corporation) were pipetted into the cup. Ten microliters of test solution were added. One milliliter of citrated blood was pipetted into the kaolin vial, capped, and gently inverted five times, after which 330 μL of the mixture was added to the TEG^®^ cup (total volume: 360 μL).

The following TEG parameters were determined at 37 °C: reaction time (R), defined as the time from initiation of the test to initial fibrin formation (2 mm amplitude), K-time (K), defined as the time measured from the beginning of clot formation (2 mm amplitude) until a fixed level of clot firmness is reached (20 mm amplitude), alpha (α), which measures the rapidity of fibrin build-up and cross-linking and is the angle formed between the horizontal at 2 mm and the tangent at 20 mm amplitude, the maximum amplitude (MA) of the TEG tracing, which is a direct function of the maximum dynamic properties of fibrin and platelet bonding via GPIIb/IIIa receptors and represents the ultimate strength of the clot, and the percent lysis 30 min post-MA (LY30), which is the percent reduction of area under the TEG tracing from MA to 30 min after MA is reached (Table S4).

### 4.3. Development of a Translational Rabbit Model of Uncontrolled Hemorrhagic Shock

After completing the ex vivo experiments in rats, we developed a clinically relevant translational rabbit model of uncontrolled hemorrhagic shock and hypotensive resuscitation to determine the resuscitative efficacy and coagulation effects of centhaquine.

#### 4.3.1. Animal Preparation

Male New Zealand rabbits (60–70 days in age; weighing 2.4–2.7 kg) were housed in individual cages on a 12-h light/dark cycle and had free access to standard feed and water for a minimum of a seven-day adaptation period before the experiment. The laboratory temperature was maintained at 24 ± 1 °C. The animals were fasted overnight but had free access to water before the experiment.

In brief, the animals in the operation research facility were anesthetized via intramuscular injection of ketamine (35 mg kg^−1^) and xylazine (5 mg kg^−1^) in the thigh muscle. The animals were immobilized on a surgical board equipped with a controlled heating pad. The core temperature measured via a rectal probe was maintained at 37 °C. Adequacy of anesthesia was monitored throughout the experiment by the loss of the ear pinch reflex, toe pinch reflex, and corneal reflex, and ensured by the absence of spontaneous movement to painful stimulation and monitoring of cardiovascular parameters of sympathetic activity (stable heart rate and arterial blood pressure).

A surgical tracheostomy was performed, and a 3.5 mm uncuffed tube (3.5 mm Portex; Smiths Medical, Kent, UK) was placed and secured into the trachea. Successful placement was ascertained by auscultation of both lungs while ventilated with a self-inflating bag. The tracheal tube was connected to a ventilator (Model 683, Harvard Apparatus Inc., Holliston, MA, USA), and the animals were mechanically ventilated (fraction of inspired oxygen: 0.21) in order to maintain constant arterial blood gases and pH and to avoid the effect of respiration on hemodynamics. Additional doses of 10 mg kg^−1^ ketamine were administered intravenously every 30 min to 45 min as required and, if needed, 1.5 mg kg^−1^ xylazine was administered intravenously every 60 min to 90 min.

Under aseptic conditions, the left jugular vein was surgically prepared and cannulated using PE tubing for the administration of resuscitative fluids and drugs. Then, the left carotid artery was surgically prepared and cannulated using PE 60 tubing, and a Mikro-Tip catheter (MPR-500 5F, Millar) was inserted to monitor blood pressure. This sensor was connected to PowerLab (AD Instruments). Intravascular catheters attached to pressure transducers were aligned to the right atrium level and calibrated before their use. The right femoral artery was also cannulated using PE 60 tubing for blood sampling. Arterial blood gases were measured using a blood gas analyzer (GEM Premier 3000, Instrumentation Laboratory, Lexington, MA, USA), while TEG analysis was performed using 1.0 mL of blood.

**Figure 9:**
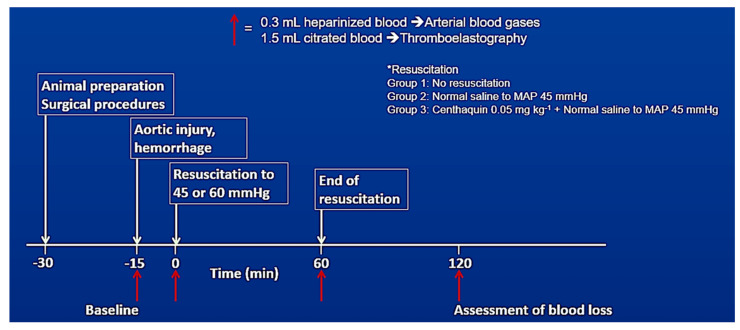
Flow chart of the experimental procedure in rabbits with uncontrolled hemorrhage.

A midline laparotomy was performed under sterile conditions, and 2-0 nylon continuous full−thickness running sutures were placed through the edges of the laparotomy for later abdominal closure. Thereafter, the abdomen was covered with a plastic film to reduce insensible fluid losses, and each rabbit was allowed to stabilize for 15 min. Respiratory and cardiovascular parameters, including electrocardiogram, were continuously monitored. The experimental protocol outline is depicted in Figure 9.

#### 4.3.2. Experimental Protocol

Baseline data, including arterial blood gases and TEG variables, were collected following the 15 min stabilization period. Hemorrhage was induced by a standardized single-puncture injury to the left side of the infrarenal aorta, two millimeters below the lower pole of the left kidney, with a 16-gauge needle. Then, the abdomen was immediately closed by pulling the previously placed sutures. After another 15 min period to simulate the time emergency medical services would take to arrive at the scene and start resuscitation, animals were randomly assigned to three treatment groups:

- Group 1: injury leading to uncontrolled hemorrhage + no resuscitation; n = 10 (no resuscitation group)
- Group 2: injury leading to uncontrolled hemorrhage + normal saline infused to maintain a MAP of 45 mmHg for 60 min; n = 10 (saline 45 mmHg group)
- Group 3: injury leading to uncontrolled hemorrhage + centhaquine (0.05 mg kg^−1^) along with normal saline to maintain a MAP of 45 mmHg for 60 min; n = 10 (centhaquine 45 mmHg group)

The infusion rate of administered fluids was kept <5 mL min^−1^ to maintain the MAP target. After the end of resuscitation, all animals were observed for an additional 60 min, while coagulation was monitored by TEG as previously described.

After the end of the observation phase, the abdomen was re-opened to measure blood loss via absorption with pre-weighed gauze pads. Total intra-abdominal blood loss was calculated as the difference between blood-soaked sponges minus the weight of dry sponges (assuming that 1 g of blood weight is equal to one milliliter of blood volume) [64,65]. As post-resuscitation clinical recovery is commonly hindered by physiological dysfunction in multiple organ systems [66], the pulmonary wet-to-dry ratio, which represents the percentage of tissue water and is an index of tissue microvascular permeability, was analyzed to examine the potential for fluid retention and pulmonary edema. Thus, the lungs were removed, and the wet weight was measured immediately after dissection. Dry weights were measured after drying specimens at 80 °C for 72 h to constant weight [67].

### 4.4. Assessment of the Effect of Hypotensive Resuscitation with Centhaquine Versus a Higher Blood Pressure Target on Blood Coagulation

In a subsequent study using the previously described rabbit model, we investigated the effect of hypotensive resuscitation with centhaquine versus a higher MAP target on blood coagulation. Male New Zealand white rabbits anesthetized and instrumented as described above were assigned to three groups:

- Group 1: injury leading to uncontrolled hemorrhage + normal saline infused to maintain a MAP of 45 mmHg; n = 13 (Sal-MAP 45 group)
- Group 2: injury leading to uncontrolled hemorrhage + centhaquine (0.05 mg kg^−1^) along with normal saline to maintain a MAP of 45 mmHg; n = 11 (Centh-MAP 45 group)
- Group 3: injury leading to uncontrolled hemorrhage + normal saline infused to maintain a MAP of 60 mmHg; n = 5 (Sal-MAP 60 group)

Resuscitation was started 15 min after the aortic puncture, as previously described. The infusion rate of administered fluids was kept <5 mL min^−1^ in the Sal-MAP 45 and Centh-MAP 45 groups, while in the Sal-MAP 60 group normal saline was infused at a higher rate to maintain MAP at 60 mmHg. Target MAPs were maintained for 60 min, while all animals were observed for an additional 60 min. Blood loss and coagulation were assessed by pre-weighed gauze pads and TEG, respectively, as previously described.

### 4.5. Statistical Analysis

#### 4.5.1. Rat Study

Data are presented as mean ± S.E. The Student’s *t*-test, with or without the Welch’s correction for unequal variances, was used to assess the statistical significance of the difference in the TEG parameters (including R, K, α, MA, and LY30). The non-parametric Mann–Whitney U test was used when the distribution was skewed. Only pairwise comparisons were used with a Bonferroni correction. An adjusted *p*-value lower than 0.05 was considered to be significant. Analyses were performed by GraphPad Prism 5.00 (GraphPad, San Diego, CA, USA).

#### 4.5.2. Rabbit Studies

An a priori power analysis was conducted using GraphPad Instat-2.00. The power was set to 80% (beta = 0.8) and the significance level (alpha) used was 0.05. The power analysis indicated that a sample size of 8 animals per group was sufficient to achieve a power of 80% when the level of significance alpha = 0.05. As hemorrhage models tend to produce great variability in mortality rate, and the overall mortality reported in rabbit models of uncontrolled hemorrhage is about 20% [64], the number of animals in each group was set at 10 for the development of the translational rabbit model of uncontrolled hemorrhagic shock (i.e., Section 4.3).

In the subsequent rabbit study (i.e., Section 4.4), the number of animals in the Sal-MAP 45 and Centh-MAP 45 groups were set at 13 and 11, respectively, for the same reason as above, i.e., because hemorrhage models tend to produce great variability in the mortality rate [64]. Considering that animals in the Sal-MAP 60 group would only undergo additional fluid administration (compared to the first rabbit study and the Sal-MAP 45 group of the second rabbit study), and taking into consideration the principles of the 3Rs (see Section 4.1), the number of animals in the Sal-MAP 60 group was set at five.

Data are presented as mean ± S.E. The significance of differences was estimated by repeated-measures analysis of variance (ANOVA) followed by a post-hoc test (Bonferroni’s method) in cases where data were collected from the same animals at different time points, and by one-way ANOVA followed by a post-hoc test (Bonferroni’s method) when comparing measurements from different animals. A p-value lower than 0.05 was considered to be significant. Analyses were performed using GraphPad Prism 10.1.2 (GraphPad, San Diego, CA, USA).

## 5. Conclusions

This is the first report of ex vivo and in vivo effects of centhaquine on blood coagulation. Centhaquine does not affect fibrin formation, platelet aggregation, and clot lysis time, nor does it alter the antithrombotic effect of aspirin and heparin in blood collected from healthy rats. Therefore, centhaquine has no direct coagulation-modulating properties under normal conditions. In uncontrolled hemorrhagic shock, centhaquine does not adversely affect blood coagulation; instead, it increases the strength of the clot and reduces blood loss and fluid requirements.

The results of the present multiphase translational study, together with those of randomized controlled trials, suggest that centhaquine is the first vasopressor agent that does not affect coagulation and may facilitate hemostatic resuscitation. These features are important for clinical practice and may represent an important breakthrough in the management of conditions other than hypovolemic shock, such as sepsis and septic shock, or in the perioperative management of surgical patients.

## Figures and Tables

**Table 1:** Effect of centhaquine, heparin, and their combination on TEG parameters in citrated whole blood from normal rats.

	R (min)	K (min)	α (°)	MA (mm)	LY30 (%)
Vehicle ^#^	2.9 ± 0.2	0.96 ± 0.0	76.0 ± 0.7	71.2 ± 0.7	0.14 ± 0.0
Centhaquine	3.8 ± 0.4	1.11 ± 0.1	74.6 ± 1.1	72.1 ± 0.4	0.22 ± 0.1
% change	23.8	13.2	−1.9	1.2	37.5
Vehicle ^#^	2.9 ± 0.2	0.96 ± 0.0	76.0 ± 0.7	71.2 ± 0.7	0.14 ± 0.0
Heparin	6.5 ± 0.7 *	2.68 ± 0.3 *	57.6 ± 3.0 *	64.2 ± 1.0 *	0.42 ± 0.3
% change	55.5	64.1	−32.0	−11.0	67.0
Heparin	6.5 ± 0.7	2.68 ± 0.3	57.6 ± 3.0	64.2 ± 1.0	0.42 ± 0.3
Centhaquine + heparin	8.2 ± 1.0	4.37 ± 1.0	46.1 ± 4.9	65.3 ± 0.9	0.02 ± 0.0
% change	21.0	38.5	−24.8	1.8	−2400

Values are expressed as mean ± S.E.M., n = 11 in the vehicle-treated group, n = 10 in centhaquine-treated group, n = 6 in heparin- and centhaquine + heparin-treated groups. ^#^ Vehicle was normal saline. * *p* < 0.005 vs. vehicle-treated group.

**Table 2:** Effect of centhaquine, aspirin, and their combination on TEG parameters in citrated whole blood from normal rats.

	R (min)	K (min)	α (°)	MA (mm)	LY30 (%)
Vehicle ^#^	2.9 ± 0.2	0.9 ± 0.1	77.9 ± 1.3	74.6 ± 1.8	0.3 ± 0.1
Centhaquine	2.6 ± 0.2	0.8 ± 0.0	79.2 ± 0.6	74.4 ± 1.4	0.1 ± 0.1
% change	−9.8	−15.0	1.7	−0.3	−100.0
Vehicle ^#^	2.9 ± 0.2	0.9 ± 0.1	77.9 ± 1.3	74.6 ± 1.8	0.3 ± 0.1
Aspirin	2.9 ± 0.1	0.8 ± 0.0	79.0 ± 0.7	64.4 ± 2.0 *	0.8 ± 0.4
% change	−0.7	−9.5	1.4	−15.8	65.0
Aspirin	2.9 ± 0.1	0.8 ± 0.0	79.0 ± 0.7	64.4 ± 2.0	0.8 ± 0.4
Centhaquine + aspirin	2.7 ± 0.3	0.8 ± 0.0	78.8 ± 0.7	65.4 ± 1.5	1.1 ± 0.5
% change	−8.3	−5.0	−0.3	1.5	28.6

Values are expressed as mean ± S.E.M., n = 5. ^#^ Vehicle was water:PEG 400 4:1 *v*/*v*. * *p* = 0.0053 vs. vehicle-treated group.

## Data Availability

Data can be made available upon request after publication through a collaborative process. Researchers should provide a methodically sound proposal with specific objectives in an approval proposal. Please contact the corresponding author for additional information.

## Supplementary Materials

The supporting information can be downloaded at: https://www.mdpi.com/article/10.3390/ijms25063494/s1.
