# Supplementary material for: Effect of Centhaquine on the Coagulation Cascade in Normal State and Uncontrolled Hemorrhage: A Multiphase Study Combining Ex Vivo and In Vivo Experiments in Different Species
Source: Int J Mol Sci. 2024 Mar 20;25(6):3494. doi: 10.3390/ijms25063494 (PMC10970680; doi:10.3390/ijms25063494)
Supplement: Supplementary file 1 [file ijms-25-03494-s001.zip › ijms-2904038-supplementary.pdf]

SUPPLEMENTAL MATERIAL

Effect of centhaquine on the coagulation cascade in normal state and uncontrolled hemorrhage: A multiphase study combining ex vivo and in vivo experiments in different species

Athanasios Chalkias [1,2 * #], Gwendolyn Pais [3 #] and Anil Gulati [3,4,5]

[1] Institute for Translational Medicine and Therapeutics, University of Pennsylvania Perelman School of Medicine, Philadelphia, PA 19104-5158, USA

[2] Outcomes Research Consortium, Cleveland, OH 44195, USA

[3] Chicago College of Pharmacy, Midwestern University, Downers Grove, IL 60515, USA

[4] Pharmazz Inc., Research and Development, Willowbrook, IL 60527, USA

[5] Department of Bioengineering, The University of Illinois at Chicago, Chicago, IL 60607, USA

SUPPLEMENTAL FIGURES

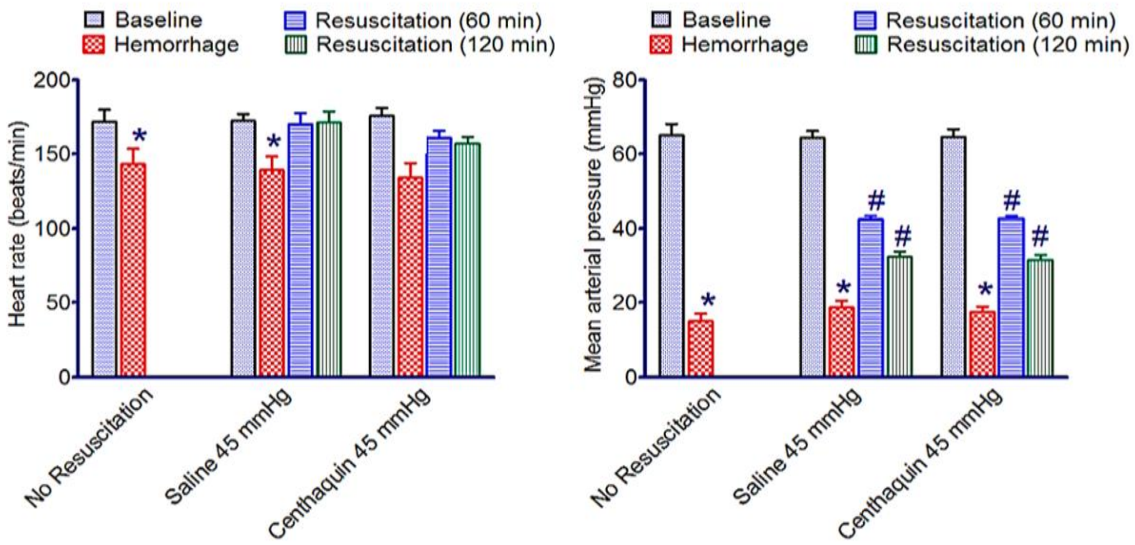

Figure S1. Differences in heart rate between groups.

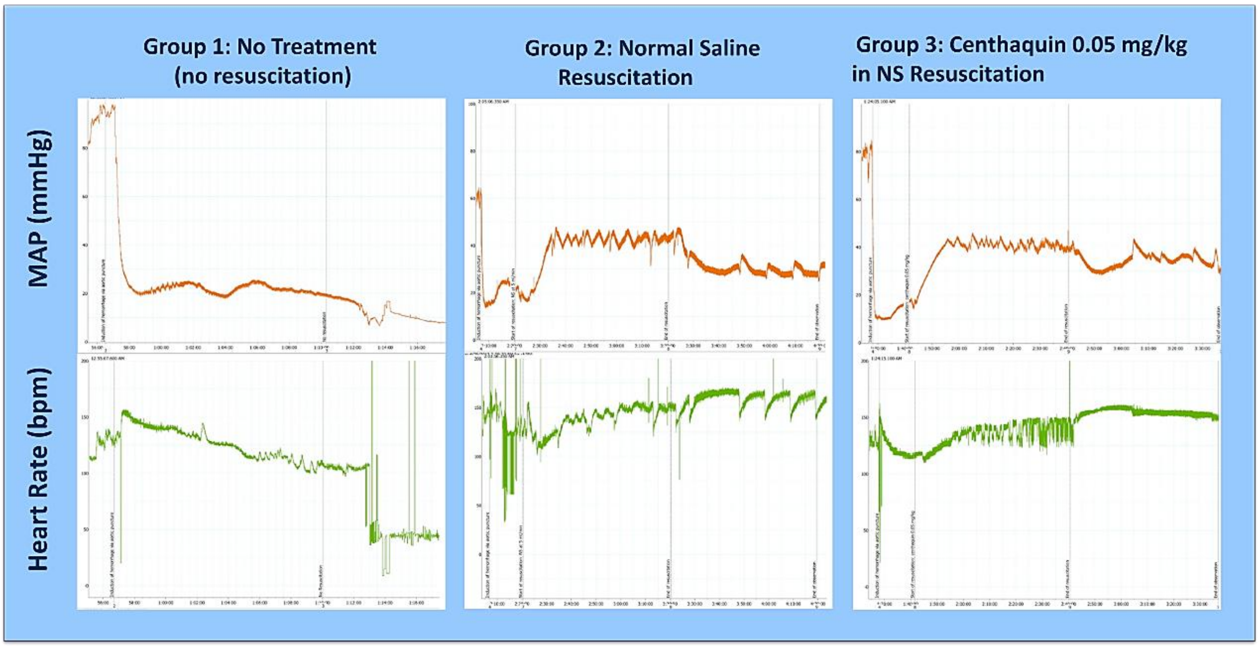

Figure S2. Trend of heart rate and mean arterial pressure during the experiment.

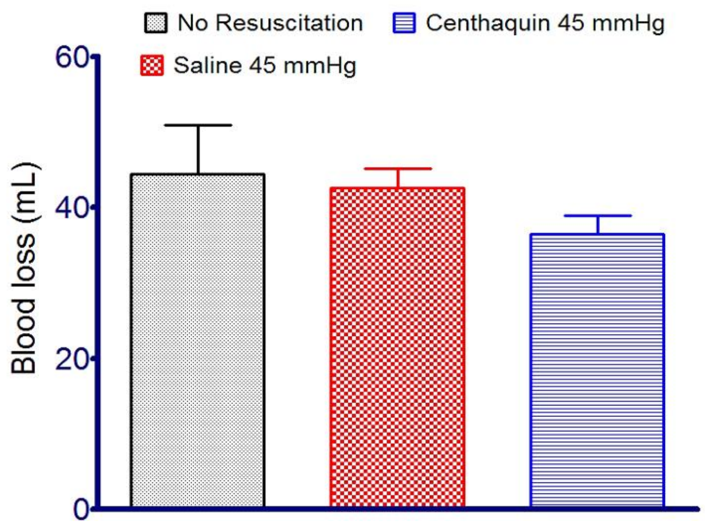

Figure S3. Differences in blood loss between groups. Blood loss was measured via absorption with pre-weighed gauze pads by re-opening the abdomen at the end of observation. Blood loss for no resuscitation animals (mean ± SEM = 44.4 ± 6.6 mL), normal saline (42.5 ± 2.5 mL), and centhaquin (36.4 ± 2.5 mL) groups was not significantly different. Centhaquin-resuscitated animals showed less blood loss than normal saline animals.

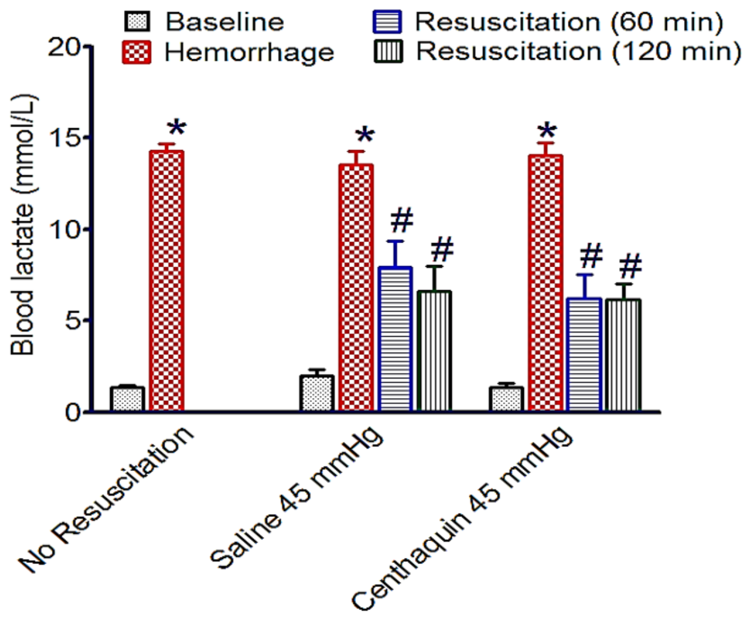

Figure S4. Lactate levels in all groups during the experiment. Across all groups, lactate levels increased significantly with hemorrhage ($p<0.05$). Resuscitation significantly decreased lactate post-hemorrhage ($p<0.05$).

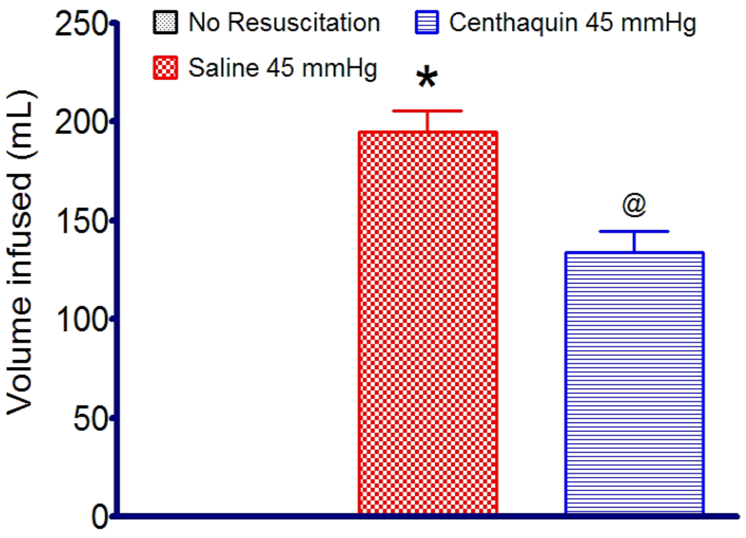

Figure S5. Differences in volume infused between groups. Animals in the centhaquin resuscitation group required significantly less infusion volume (133.9 ± 10.8 mL) compared to normal saline animals (195.1 ± 10.7 mL) to maintain mean arterial pressure at 45 mmHg for one hour (p<0.001).

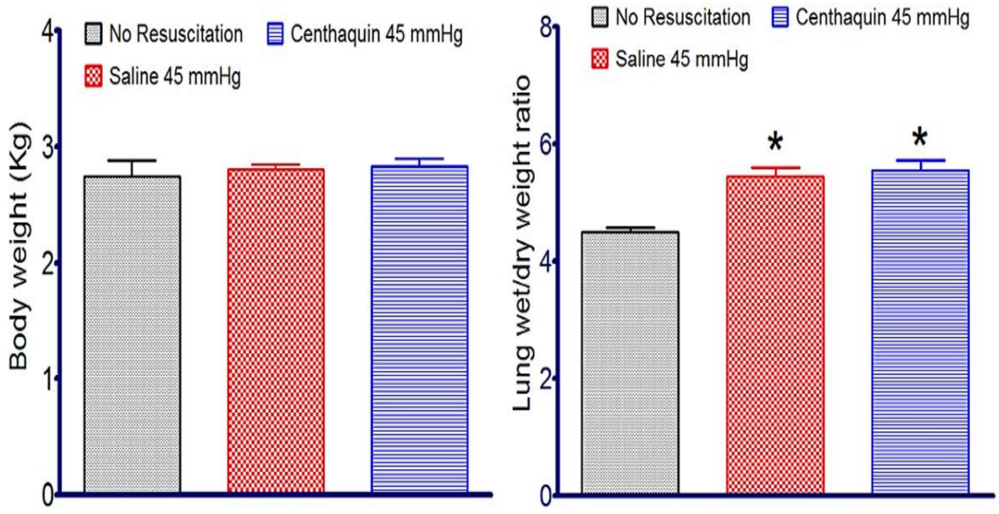

Figure S6. Differences in body weight between groups. Body weight was nearly identical across groups (no resuscitation 2.7 ± 0.08 kg; normal saline 2.7 ± 0.05 kg; centhaquin 2.8 ± 0.06 kg. The wet to dry ration for the normal saline group (5.45 ± 0.15) and the centhaquin group (5.56 ± 0.16) did not significantly differ, but were both significantly greater than the ratio for no resuscitation animals (4.50 ± 0.08) (p=0.0028).

SUPPLEMENTAL TABLES

Table S1. Change of thromboelastography parameters in heparin *vs.* vehicle and aspirin *vs.* 0.3% DMSO

% change	R (min)	K (min)	α (°)	MA (mm)	LY30 (%)
**Heparin *vs.* Vehicle**	55.5	64.1	-32.0	-11.0	67.0
**Aspirin *vs.* 0.3% DMSO**	-1.6	0.0	-0.4	-10.8	-24.7

Table S2. Hemodynamic changes during the experiment

	Heart rate				Mean arterial pressure			
	Baseline	Hemorrhage	Resuscitation (60 min)	Resuscitation (120 min)	Baseline	Hemorrhage	Resuscitation (60 min)	Resuscitation (120 min)
**No resuscitation**	171.7 ± 8.3	143.2 ± 10.6	–	–	64.6 ± 2.1	15.1 ± 1.9	–	–
**Normal saline**	172.3 ± 4.4	139.1 ± 9.2	170.0 ± 7.9	171.0 ± 7.7	64.5 ± 1.7	18.8 ± 1.9	42.4 ± 1.0	32.4 ± 1.3
**Centhaquine**	175.8 ± 5.4	134.1 ± 9.9	160.9 ± 4.9	156.8 ± 4.6	64.6 ± 2.1	17.6 ± 1.3	42.7 ± 0.6	31.6 ± 1.2

Values expressed as mean ± SEM.

Table S3. Changes in lactate levels during the experiment

	Baseline	Hemorrhage	Resuscitation (60 min)	Resuscitation (120 min)
**No resuscitation**	1.37 ± 0.12	14.23 ± 0.42	–	–
**Normal saline**	2.03 ± 0.31	13.53 ± 0.75	7.93 ± 1.46	6.65 ± 1.34
**Centhaquine**	1.34 ± 0.15	14.04 ± 0.67	6.2 ± 1.35	6.14 ± 0.87

Values expressed as mean ± SEM.

Table S4. Thromboelastography parameters

**Hemostasis parameter**	**TEG parameter**	**Measurement**
Enzymatic portion of coagulation	R, reaction time (min)	The period of time of latency from the time that the blood was placed in the TEG analyzer until initial fibrin formation i.e., till an amplitude of 2 mm.
Clot kinetics	K (min)	Measures the speed to reach a certain level of clot strength i.e., time from 2 mm to 20 mm amplitude.
Fibrinogen level	α (°)	Measures the rapidity of fibrin build-up and cross-linking. It is the angle formed between the horizontal at 2 mm and the tangent at 20 mm amplitude
Platelet function/aggregation	MA, maximum amplitude (mm)	A direct function of the maximum dynamic properties of fibrin and platelet bonding via GPIIb/IIIa receptors and represents the ultimate strength of the clot
Clot lysis	LY30 (%)	Measures the rate of amplitude reduction 30 min after MA. It is the reduction of the area under the curve from the time MA is measured until 30 min after MA.
